# P-1130. Promoting De-Implementation of Inappropriate Antimicrobial Use Following Cardiac Device Procedures

**DOI:** 10.1093/ofid/ofaf695.1324

**Published:** 2026-01-11

**Authors:** Judith Strymish, Rebecca P Lamkin, Hillary J Mull, Anna Chen, Marlena Shin, Samuel Golenbock, Dipandita Basnet Thapa, Dimitri M Drekonja, Huan Xu, Kathryn L Colborn, Maria C Rodriguez-Barradas, Westyn Branch-Elliman

**Affiliations:** VA Boston Healthcare System, West Roxbury, MA; VA Boston Healthcare System, West Roxbury, MA; VA Boston Healthcare System, West Roxbury, MA; VA Boston HCS, Boston, Massachusetts; VA Boston Healthcare System, West Roxbury, MA; VA Boston Healthcare System, West Roxbury, MA; VA Boston Healthcare System, West Roxbury, MA; Minneapolis VA Health Care System, Minneapolis, Minnesota; 4VA Houston, houston, Texas; University of Colorado, Aurora, Colorado; Michael E. DeBakey VAMC and Baylor College of Medicine, Houston, Texas; Greater Los Angeles VA Healthcare System, UCLA David Geffen School of Medicine, Los Angeles, CA

## Abstract

**Background:**

Despite a strong evidence base and clinical guidelines specifcally recommending against prolonged post-procedural antimicrobial use, studies indicate that the practice is common following cardiac device (CIED) procedures, including the VA. The aim of this study was to leverage learning/unlearning theory and de-implementation science to promote uptake of guideline-based care and to reduce inappropriate antimicrobial prophylaxis following CIED procedures.

Formative evaluations conducted by our VA study team suggested that inappropriate antimicrobial use is driven by several factors including concerns about liability if post-procedural antimicrobials are not prescribed (Figure 1).

**Methods:**

We conducted a hybrid type III effectiveness-implementation stepped-wedge intervention trial of a bundled de-implementation intervention at three high-volume, high-complexity VA medical centers. The bundled de-implementation intervention included: audit and feedback with benchmarking about guideline concordant care, CIED infection, and antimicrobial harms, education, engagement of local champions, and blended facilitation. Primary outcome was facility uptake of guideline-concordant care. Intervention sites were compared to three matched controls. Qualitative interviews were used to determine factors associated with practice improvement (Figure 2).

**Results:**

Facility rates of guideline discordant practice at the 3 intervention and 3 control sites are presented in Figures 3; days of post-procedural antibiotics are presented in Figure 4.. Two sites demonstrated substantial improvement, with reductions in guideline discordant care from >90% of procedures to < 15% of procedures. In the third site, rates of prolonged prophylaxis remained high but median duration of antimicrobial prophylaxis was reduced. No increases in CIED infections following discontinuation were identified. No improvements were seen in the non-intervention sites. Practice changes were sustained for a least 1 year.

**Conclusion:**

A multifaceted de-implementation bundle that includes infection surveillance to provide assurance that patients are not being harmed by reduced antimicrobial exposure was effective at promoting guideline uptake and reducing inappropriate antimicrobial use.

**Disclosures:**

Westyn Branch-Elliman, MD, MMSc, DLA Piper, LLC/Medtronic: Advisor/Consultant|DLA Piper, LLC/Medtronic: Expert Testimony

